# Vertebrate ancient opsin photopigment spectra and the avian photoperiodic response

**DOI:** 10.1098/rsbl.2011.0864

**Published:** 2011-10-26

**Authors:** Wayne I. L. Davies, Michael Turton, Stuart N. Peirson, Brian K. Follett, Stephanie Halford, Jose M. Garcia-Fernandez, Peter J. Sharp, Mark W. Hankins, Russell G. Foster

**Affiliations:** 1 Nuffield Laboratory of Ophthalmology, Oxford University, Oxford OX3 9DU, UK; 2 Department of Zoology, Oxford University, Oxford OX1 3PS, UK; 3 Departamento de Morfología y Biología Celular Facultad de Medicina, Oviedo Universidad, Oviedo 33006, Spain; 4 The Roslin Institute, Edinburgh University, Edinburgh EH25 9PS, UK

**Keywords:** avian, deep brain photoreceptor, hypothalamus, vertebrate ancient opsin, photoperiodic response

## Abstract

In mammals, photoreception is restricted to cones, rods and a subset of retinal ganglion cells. By contrast, non-mammalian vertebrates possess many extraocular photoreceptors but in many cases the role of these photoreceptors and their underlying photopigments is unknown. In birds, deep brain photoreceptors have been shown to sense photic changes in daylength (photoperiod) and mediate seasonal reproduction. Nonetheless, the specific identity of the opsin photopigment ‘sensor’ involved has remained elusive. Previously, we showed that vertebrate ancient (VA) opsin is expressed in avian hypothalamic neurons and forms a photosensitive molecule. However, a direct functional link between VA opsin and the regulation of seasonal biology was absent. Here, we report the *in vivo* and *in vitro* absorption spectra (*λ*_max_ = ∼490 nm) for chicken VA photopigments. Furthermore, the spectral sensitivity of these photopigments match the peak absorbance of the avian photoperiodic response (*λ*_max_ = 492 nm) and permits maximum photon capture within the restricted light environment of the hypothalamus. Such a correspondence argues strongly that VA opsin plays a key role in regulating seasonal reproduction in birds.

## 1. Introduction

Birds possess photoreceptors located within the medio-basal hypothalamus (MBH) that regulate photoperiodic responses to daylength [1]. An action spectrum for this response showed the involvement of an opsin/vitamin A photopigment with a spectral maximum at 492 nm, suggesting that daylength measurements are mediated by such a molecule [2]. However, the specific molecular identity of this opsin has remained ambiguous, with rod-like opsin [3], melanopsin [4], neuropsin (OPN5) [5] and vertebrate ancient (VA) opsin [6] all being proposed as potential candidates. Traditionally, a number of criteria must be satisfied before assigning a particular function to a photopigment. Specifically, the molecular candidate should: (i) be expressed in photoreceptors/photosensitive areas; (ii) form a functional photopigment; and (iii) possess an absorption spectrum with a spectral maximum (*λ*_max_) that matches the action spectrum for the biological response. An additional supportive, association (iv) is whether the candidate photopigment is spectrally ‘tuned’ to the prevailing light conditions, as often seen in the visual pigments driving colour vision or the rod photopigments of deep-sea fishes. Based upon criteria (i) and (ii), VA opsin [6] and OPN5 [5] have emerged as the strongest candidates. However, their match to criteria (iii) and (iv) is problematic as information is either confounding or lacking. In the case of chicken OPN5 (cOPN5), absorbance spectroscopy has demonstrated a photopigment with a *λ*_max_ at 360 nm [7]. Such a spectral maximum would not support cOPN5 as the photoperiodic photopigment, based upon the finding that the action spectrum for the avian photoperiodic response peaks at 492 nm [2] and the observation that little ultraviolet (UV) light can penetrate tissue [8].

Chicken VA (cVA) opsin encodes two isoforms, cVALong (cVAL) and cVAShort (cVAS), that both form functional photopigments with cVAS protein being detected within the hypothalamus [6]. Until now, the absorption spectra for these photopigments were not known, so any potential match between action and absorption spectra could not be evaluated. This paper addresses this issue by reporting the absorption spectra for both chicken VA isoforms and assessing whether their absorbance characteristics are appropriately tuned to the spectral composition of light penetrating the avian hypothalamus.

We find that cVAS and cVAL exhibit a *λ*_max_ at approximately 490 nm, differing by only 2 nm from the peak of the action spectrum for the avian photoperiodic response at 492 nm. Furthermore, because of the absorbance characteristics of haemoglobin, there is a small ‘spectral window’ in hypothalamic light penetration that peaks around 489 nm. Thus, the spectral maxima of cVA photopigments are ideally suited to maximize photon capture within the bird hypothalamus. These new findings allow us to satisfy criteria (iii) and (iv) of assigning a candidate photopigment to a physiological function, namely, that VA opsin plays a central role in regulating the avian photoperiodic response.

## 2. Material and methods

Hypothalamic lysates from dark-adapted chickens were homogenized in phosphate buffered saline/n-dodecyl-β-d-maltoside/phenylmethylsulphonylfluoride buffer (pH 7.4) [9] under dim red light. Native cVAS photopigments, containing endogenous 11-*cis* retinal, were extracted in the dark by affinity immunochromatography [9] by passing the hypothalamic lysates through a cyanogen bromide-sepharose binding column coupled to an anti-cVA antibody [6]. For recombinant expression experiments, cVAL was transfected into HEK293T cells and incubated at 30°C or 37°C. Post-harvesting, membrane fractions were incubated with excess 11-*cis* retinal and cVAL photopigments purified by affinity immunochromatography [9]. All absorbance spectra (dark and bleached) were recorded in triplicate using a Shimadzu UV-visible spectrophotometer, with difference spectra being calculated prior to template fitting to minimize the spectral distortion of underlying absorbance and scatter of the protein [10]. The spectral peak absorbance (*λ*_max_) and coefficient of determination (*r*^2^ value) were determined by fitting these data around the peak and long-wavelength limb of a modified Govardovskii template [11] with a subtracted retinal oxime spectrum [10] using an Excel spreadsheet with a Solver function by the method of least squares [9].

## 3. Results

**Figure 1.**
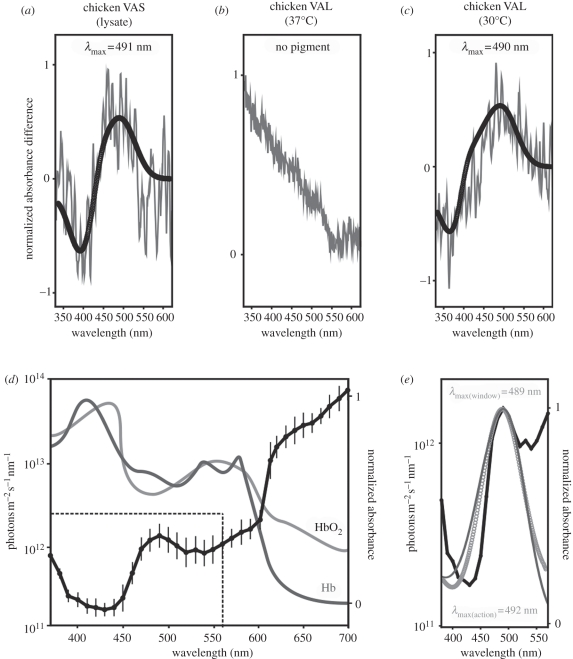
(*a*) Absorbance difference spectra (grey line) for cVAS opsin photopigments extracted from hypothalamic lysates and (*b*) HEK293T cells transfected with cVAL and incubated at 37°C or (*c*) 30°C. *λ*_max_ values are shown, as determined by fitting to a modified Govardovskii template [10,11] (black circles). (*d*) Spectral composition of sunlight reaching the basal hypothalamus (modified from [12]; black line) compared with the absorbance of oxygenated (HbO_2_; light grey line) and deoxygenated (Hb; dark grey line) haemoglobin (modified from [13]). Dotted box refers to the spectrum highlighted in (*e*), analysed within a physiological range for vitamin A_1_-based photopigments (*λ*_max_ = 360–560 nm). (*e*) Spectrum of light penetrance at the avian hypothalamus (black line; modified from [12]), showing a ‘spectral window’ with a *λ*_max_ at 489 nm when fitted to a vitamin A_1_-based template (light grey circles), compared with the action spectrum for the avian photoperiodic response (dark grey line) with a *λ*_max_ at 492 (modified from [2]).

Chicken VA exists as two isoforms (cVAL and cVAS), with Western blot analysis showing that cVAS is the only expressed VA isoform detected in the hypothalamus [6]. Therefore, we determined the spectral sensitivity of this photopigment by extracting *in vivo* protein from dark-adapted chicken hypothalamic lysates to ensure that the native protein remained conjugated to its endogenous 11-*cis* retinal chromophore. As shown in figure 1*a*, UV-visible spectrophotometry yielded a cVAS photopigment spectrum with a *λ*_max_ at 491 nm (*r*^2^ = 0.5). The spectral characteristics of cVAL were determined by *in vitro* recombinant expression in HEK293T cells (at 37°C), followed by reconstitution with 11-*cis* retinal and UV-visible spectrophotometry. Despite repeated attempts, characteristic photopigment-like difference spectra were not obtained (figure 1*b*). These experiments were then repeated at 30°C. Our rationale was that the lower temperature would reduce the rate of translation and endocytic membrane clearance and provide more time for correct protein folding and trafficking of viable opsin to the cell membranes. This approach was successful and yielded a cVAL photopigment with a *λ*_max_ at 490 nm (*r*^2^ = 0.7; figure 1*c*), which was strikingly similar to the *in vivo* spectral absorbance peak of cVAS. Similar *in vitro* expression of cVAS did not successfully regenerate (data not shown), suggesting that variation in the length and sequence of the carboxyl-termini may result in protein isoforms with different folding properties. These results are consistent with previous findings that show recombinant short isoforms of vertebrate photopigments do not easily regenerate [14]. Collectively, both *in vivo* and *in vitro* approaches yielded cVA photopigments with absorbance maxima at approximately 490 nm, which only differs by 2 nm from the reported 492 nm peak of the action spectrum for the avian photoperiodic response [2]. Thus, criterion (iii) for assigning a particular function to a candidate photopigment has been satisfied.

The avian cranium is permeable to light across the visible spectrum, but scatter and photon absorption by overlying tissues significantly modifies the spectral composition of sunlight that penetrates into the hypothalamus of birds [12]. Specifically, as sunlight passes through the feathers, skin, skull and brain, wavelengths of 400–450 nm and 525–550 nm are greatly attenuated owing to the presence of haemoglobin with absorbance peaks at 430 and 550 nm (figure 1*d*) [13]. However, there is a small ‘spectral window’ in haemoglobin absorption and light penetrates into the brain between 450 and 550 nm [12], with a peak at 489 nm (*r*^2^ = 0.9; figure 1*d*,*e*). This value is significant as it closely corresponds to the *λ*_max_ for both VA photopigment isoforms (figure 1*a*,*c*) and the action spectrum of the photoperiodic response (figure 1*e*). Photons at wavelengths beyond 590 nm penetrate into the hypothalamus with greater efficiency than 489 nm (figure 1*d*) and so the question arises why not evolve long-wavelength-shifted photopigments to maximize photon capture? Such a task, however, presents several problems. The first relates to thermal noise. The more long-wavelength-shifted the *λ*_max_ of a photopigment the greater the chance of thermal isomerization [15]. As a result, distinguishing low levels of light from signals generated by heat within the 40°C environment of the deep brain would represent a significant sensory challenge. The second problem relates to the construction of a photopigment with a *λ*_max_ > 590 nm. Photopigments are conjugated to either vitamin A_1_- or A_2_-based chromophores, with the latter allowing significantly greater sensitivity to longer wavelengths compared with the former. Birds, however, are unable to manufacture vitamin A_2_ retinal, so the most long-wavelength-shifted *λ*_max_ found is approximately 560 nm. Such a *λ*_max_ would confer no additional sensitivity to light within the deep brain and be subject to greater levels of thermal noise (figure 1*d*). The supportive criterion (iv), namely that the absorbance characteristics of the VA opsin photopigment should be appropriately matched to the spectral qualities of the available light within the hypothalamus for maximal photon capture, therefore, is also fulfilled. Of final significance is that the *λ*_max_ of VA opsins from non-avian species characterized to date yield values between 505 and 510 nm [16], providing additional evidence that cVA is spectrally tuned to 490 nm.

## 4. Discussion

The first evidence of vertebrate deep brain photoreceptors emerged with studies that showed light-induced colour changes in the skin of the minnow (*Phoxinus laevis*) occurred in the absence of the eyes and pineal complex [17]. The receptor was localized to the basal brain by lesion studies that blocked the melanophore light response and it was proposed that the ependyma of the ventricles were photosensitive [17]. Subsequently, the presence of deep brain photoreceptors were demonstrated in all non-mammalian vertebrates [18,19]. Avian encephalic photoreceptors were first discovered when illumination with artificial daylengths, via glass rods placed within the hypothalami of ducks, showed that spring-like daylengths stimulated testicular growth while short winter photoperiods had no effect upon reproduction [20]. Subsequently, it was confirmed that the photoperiodic control of reproduction was mediated by extraretinal and extrapineal photoreceptors [21], specifically those that resided within the MBH [22]. Despite this long history, the characterization of such photoreceptors has been difficult. Previously, we demonstrated that the chicken *VA* opsin gene transcribes alternative splice-variants that encode two functional protein isoforms, cVAL and cVAS. Of these isoforms, cVAS is expressed within a population of hypothalamic neurons with extensive projections to the median eminence [6]. However, a functional link between these neurons and the avian photoperiodic response was elusive. In the present study, we report that the absorbance spectra for both cVAS (*in vivo*) and cVAL (*in vitro*) photopigments have a *λ*_max_ at approximately 490 nm, showing a strong correlation to the action spectrum for the avian photoperiodic response that peaks at 492 nm. In addition, we show that cVA appears to be spectrally tuned to the light environment of the hypothalamus. Collectively, our results build an overwhelming *prima facie* case for the involvement of VA opsin in the avian photoperiodic system. Whether other secondary photopigments might also contribute to this response remains an intriguing and unresolved issue.

## References

[1] Benoit J. 1964 The role of the eye and of the hypothalamus in the photostimulation of gonads in the duck. Ann. NY Acad. Sci. 117, 204–216 10.1111/j.1749-6632.1964.tb48175.x (doi:10.1111/j.1749-6632.1964.tb48175.x) 14196641

[2] Foster R. G. Follett B. K. Lythgoe J. N. 1985 Rhodopsin-like sensitivity of extra-retinal photoreceptors mediating the photoperiodic response in quail. Nature 313, 50–52 10.1038/313050a0 (doi:10.1038/313050a0) 3965970

[3] Wang G. Wingfield J. C. 2011 Immunocytochemical study of rhodopsin-containing putative encephalic photoreceptors in house sparrow, *Passer domesticus*. Gen. Comp. Endocrinol. 170, 589–596 10.1016/j.ygcen.2010.11.014 (doi:10.1016/j.ygcen.2010.11.014) 21118688

[4] Kang S. W. Leclerc B. Kosonsiriluk S. Mauro L. J. Iwasawa A. El Halawani M. E. 2010 Melanopsin expression in dopamine-melatonin neurons of the premammillary nucleus of the hypothalamus and seasonal reproduction in birds. Neuroscience 170, 200–213 10.1016/j.neuroscience.2010.06.082 (doi:10.1016/j.neuroscience.2010.06.082) 20620198

[5] Nakane Y. Ikegami K. Ono H. Yamamoto N. Yoshida S. Hirunagi K. Ebihara S. Kubo Y. Yoshimura T. 2010 A mammalian neural tissue opsin (opsin 5) is a deep brain photoreceptor in birds. Proc. Natl Acad. Sci. USA 107, 15 264–15 268 10.1073/pnas.1006393107 (doi:10.1073/pnas.1006393107) PMC2930557 20679218

[6] Halford S. 2009 VA opsin-based photoreceptors in the hypothalamus of birds. Curr. Biol. 19, 1396–1402 10.1016/j.cub.2009.06.066 (doi:10.1016/j.cub.2009.06.066) 19664923

[7] Yamashita T. Ohuchi H. Tomonari S. Ikeda K. Sakai K. Shichida Y. 2010 Opn5 is a UV-sensitive bistable pigment that couples with Gi subtype of G protein. Proc. Natl Acad. Sci. USA 107, 22 084–22 089 10.1073/pnas.1012498107 (doi:10.1073/pnas.1012498107) 21135214 PMC3009823

[8] Lewis P. D. Gous R. M. 2009 Responses of poultry to ultraviolet radiation. World's Poult. Sci. J. 65, 499–510 10.1017/S0043933909000361 (doi:10.1017/S0043933909000361)

[9] Davies W. I. Zheng L. Hughes S. Tamai T. K. Turton M. Halford S. Foster R. G. Whitmore D. Hankins M. W. In press Functional diversity of melanopsins and their global expression in the teleost retina. Cell Mol. Life Sci. 10.1007/s00018-011-0785-4 (doi:10.1007/s00018-011-0785-4) PMC11114754 21833582

[10] Parry J. W. Poopalasundaram S. Bowmaker J. K. Hunt D. M. 2004 A novel amino acid substitution is responsible for spectral tuning in a rodent violet-sensitive visual pigment. Biochemistry 43, 8014–8020 10.1021/bi049478w (doi:10.1021/bi049478w) 15209496

[11] Govardovskii V. I. Fyhrquist N. Reuter T. Kuzmin D. G. Donner K. 2000 In search of the visual pigment template. Vis. Neurosci. 17, 509–528 10.1017/S0952523800174036 (doi:10.1017/S0952523800174036) 11016572

[12] Foster R. G. Follett B. K. 1985 The involvement of a rhodopsin-like photopigment in the photoperiodic response of the Japanese quail. J. Comp. Physiol. A 157, 519–528 10.1007/BF00615153 (doi:10.1007/BF00615153)

[13] Antonini E. Brunori M. 1971 Haemoglobin and myoglobin in their reactions with ligands. In Frontiers of biology (eds Neuberger A. Tatum E. L.), pp. 13–54 Amsterdam, The Netherlands: North Holland

[14] Kojima D. Mano H. Fukada Y. 2000 Vertebrate ancient-long opsin: a green-sensitive photoreceptive molecule present in zebrafish deep brain and retinal horizontal cells. J. Neurosci. 20, 2845–2851 10751436 10.1523/JNEUROSCI.20-08-02845.2000 PMC6772192

[15] Barlow H. B. 1957 Purkinje shift and retinal noise. Nature 179, 255–256 10.1038/179255b0 (doi:10.1038/179255b0) 13407693

[16] Kojima D. Torii M. Fukada Y. Dowling J. E. 2008 Differential expression of duplicated VAL-opsin genes in the developing zebrafish. J. Neurochem. 104, 1364–1371 10.1111/j.1471-4159.2007.05093.x (doi:10.1111/j.1471-4159.2007.05093.x) 18036148 PMC2702163

[17] Frisch K. v. 1911 Beitrage zur Physiologie der Pigmentzellen in der Fischhaut. Pfluger's Archv Gesamte Physiol Menschen Tiere 138, 319–387 10.1007/BF01680752 (doi:10.1007/BF01680752)

[18] Young J. Z. 1935 The photoreceptors of lampreys. II. The functions of the pineal complex. J. Exp. Biol. 12, 254–270

[19] Shand J. Foster R. G. 1999 The extraretinal photoreceptors of non-mammalian vertebrates. In Adaptive mechanisms in the ecology of vision (eds Archer S. N. Djamgoz M. B. A. Loew E. R. Partridge J. C. Vallerga S.), pp. 197–222 Dordrecht, The Netherlands: Kluwer Academic Publishers

[20] Benoit J. 1935 Stimulation par la lumière artificielle du développement testiculaire chez des canards aveugles par section du nerf optique. C. R. Seances Soc. Biol. Filiales 120, 133–136

[21] Menaker M. Underwood H. 1976 Extraretinal photoreception in birds. Photophysiology 23, 299–306 1273098 10.1111/j.1751-1097.1976.tb07251.x

[22] Oliver J. Jallageas M. Bayle J. D. 1979 Plasma testosterone and LH levels in male quail bearing hypothalamic lesions or radioluminous implants. Neuroendocrinology 28, 114–122 10.1159/000122851 (doi:10.1159/000122851) 431774

